# Supplementary material for: Identification of photoperiod-induced specific miRNAs in the adrenal glands of Sunite sheep (Ovis aries)
Source: Front Vet Sci. 2022 Jul 22;9:888207. doi: 10.3389/fvets.2022.888207 (PMC9354845; doi:10.3389/fvets.2022.888207)
Supplement: Supplementary file 1 [file Data_Sheet_1.ZIP › Supplementary file/miRDeep2 file.zip!/miRDeep2 file/Novel_Structure/SP42A1/Novel_43.pdf]

Provisional ID	:	NC_019460.2_6894
Score total	:	14.4
Score for star read(s)	:	3.9
Score for read counts	:	6.8
Score for mfe	:	2.1
Score for randfold	:	1.6
Score for cons. seed	:	
Total read count	:	25
Mature read count	:	24
Loop read count	:	0
Star read count	:	1

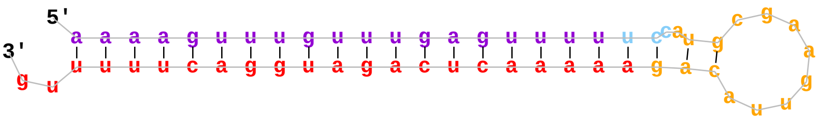

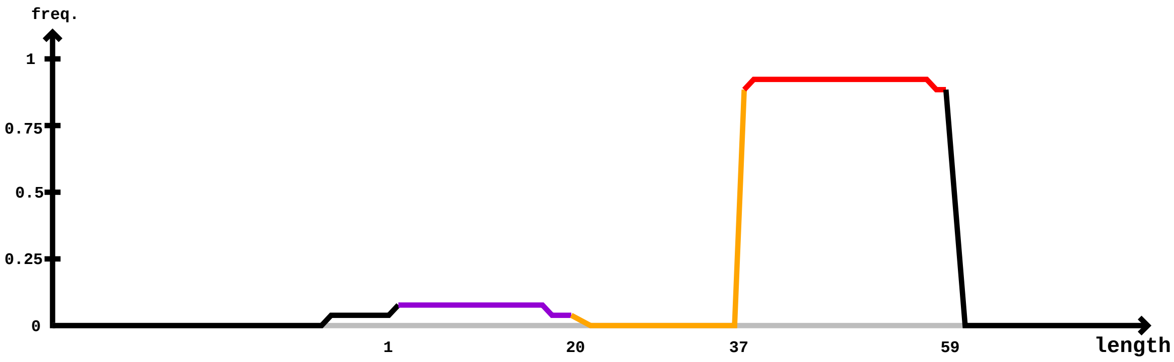


```
                                   Star                                    Mature
5'- gcauaaugacugucacaacauauugaguuggccaaaaaguuuguuugaguuuuuccaugcgaaguuacagaaaaacucagauggacuuuuuggccaacccaauauauuguac   -3'   obs
    gcauaaugacugucacaacauauugaguuggccaaaaaguuuguuugaguuuuuccaugcgaaguuacagaaaaacucagauggacuuuuuggccaacccaauauauuguac         exp
    ..............((((.((((((.(((((((((((((((((((((((((((((..((........))))))))))))))))))))))))))))))).)))))).))))..   reads   mm   sample
    ...........................uuggccaaaaaguuuAuuugagu..............................................................   1       1    seq
    ..................................aaaaguuCguuugaguuuu...........................................................   1       1    seq
    ......................................................................aaaaacucagauggacuuuuug....................   1       0    seq
    ......................................................................aaaaacucagaugAacuuuuug....................   22      1    seq
    .......................................................................aaaacucagaugAacuuuu......................   1       1    seq
```
